# Dynamic Marine Atmospheric Corrosion Behavior of AZ91 Mg Alloy Sailing from Yellow Sea to Western Pacific Ocean

**DOI:** 10.3390/ma17102294

**Published:** 2024-05-13

**Authors:** Lihui Yang, Cong Liu, Ying Wang, Xiutong Wang, Haiping Gao

**Affiliations:** 1 Key Laboratory of Advanced Marine Materials, Key Laboratory of Marine Environmental Corrosion and Bio-Fouling, Institute of Oceanology, Chinese Academy of Sciences, Qingdao 266071, China; 2 National Key Laboratory of Marine Corrosion and Protection, Luoyang Ship Material Research Institute, Qingdao 266237, China; 3 Southwest Technology and Engineering Research Institute, Chongqing 400039, China; 4 Guangxi Key Laboratory of Marine Environmental Science, Institute of Marine Corrosion Protection, Guangxi Academy of Sciences, Nanning 530007, China

**Keywords:** Mg alloy, dynamic atmospheric corrosion, sea voyage, Western Pacific Ocean

## Abstract

In this work, the dynamic marine atmospheric corrosion behavior of AZ91 Mg alloy sailing from Yellow Sea to Western Pacific Ocean was studied. The corrosion rates were measured using the weight loss method. The microstructure, phase, and chemical composition of corroded samples were investigated by SEM, EDS, XRD, and XPS. The results show that the evolution of corrosion rates of AZ91 Mg alloy was divided into three stages: rapidly increasing during the first 3 months, then remaining stable for the next three months, and finally decreasing after 6 months. The annual corrosion rate of Mg alloy reached 32.50 μm/y after exposure for 12 months in a dynamic marine atmospheric environment, which was several times higher than that of the static field exposure tests. AZ91 magnesium alloy was mainly subjected to localized corrosion with more destructiveness to Mg parts, which is mainly due to the synergistic effect of high relative humidity, the high deposition rate of chloride ion, sulfur dioxide acidic gas produced by fuel combustion, and rapid temperature changes caused by the alternating changes in longitude and latitude during navigation. As the exposure time increased, the corrosion pits gradually increased and deepened. The maximum depth of the corrosion pit was 197 μm after 12 months of exposure, which is almost 6 times the average corrosion depth. This study provides scientific data support for the application of magnesium alloys in shipborne aircraft and electronic equipment. The results could provide guidance for the design of new magnesium alloys and development of anti-corrosion technologies.

## 1. Introduction

Magnesium alloys, which display excellent properties such as low density and high specific strength, have been widely used in aerospace, automobile, and 3C industries [1,2,3]. However, the application and development of magnesium alloys are limited owing to their poor corrosion resistance, especially in harsh corrosive environments [4,5].

In order to accelerate the evaluation of the corrosion resistance of magnesium alloys, there have been a large number of studies simulating the corrosion behavior of magnesium alloys, including immersion tests and salt spray experiments [6,7,8,9,10]. However, the corrosion behavior of Mg alloys in actual service atmospheric environments is inconsistent with that in the accelerated simulated corrosion tests mentioned above [10,11,12]. It is well known that Mg alloys are mainly exposed to atmospheric environments during practical application. In recent years, many studies have been conducted on the corrosion behavior of magnesium alloys in marine and industrial environments [13,14,15,16,17,18,19,20]. Liao et al. [12] found that the corrosion rate of AZ31B in the marine atmospheric environment (Shimizu, Japan) was much higher than that in urban areas (Osaka, Japan). Jönsson et al. [14] researched the corrosion behavior of AZ91D exposed to three typical types of atmospheric environment, namely marine, rural, and urban atmospheric environments. The result showed that the corrosion rate of AZ91D exposed in the marine atmospheric environment was about 2 times higher than that in the rural and urban atmospheric environments. Yu et al. [15] studied the average corrosion rates of AM60, ZE41, AZ91D, and pure Mg exposed on an island, and found all were higher than those of samples exposed on a site located 1000 m inland from the coast (Xiamen, China). Jiang et al. [16] found that AZ91D was more susceptible to corrosion in marine environments (Qingdao, China) than in an inland environment (Beijing, China) owing to the sea salt and the high relative humidity. These studies consistently indicated that magnesium alloys suffered from more severe corrosion in marine atmospheric environments compared to inland areas.

As mentioned above, many studies of the marine atmospheric corrosion behavior of magnesium alloys have been performed in static exposure stations.

However, the service environments of magnesium alloy are dynamic when applied in a marine equipment, especially naval aviation equipment, which may lead to different corrosion behaviors compared to those in static environments. Until now, only two studies from our institute have reported the corrosion behavior of AZ31 and rare earth magnesium alloys in a dynamic marine atmosphere [17,18]. Magnesium alloys exhibited varying corrosion behaviors due to their different composition. Therefore, it is necessary to increase the amount of research on different types of magnesium alloys in dynamic marine environments. It is significant to clarify the dynamic corrosion mechanism under the interaction of multiple factors to improve the safe surface performance of Mg alloy components.

The aim of this work was to study the corrosion behavior of AZ91 Mg alloy in a dynamic marine atmospheric environment from Yellow Sea to Western Pacific Ocean. The evolution of corrosion rates, corrosion product composition, corrosion morphology, and the effects of the dynamic marine atmospheric environment on corrosion behavior of AZ91 magnesium alloy were examined. Relevant research can clarify the corrosion mechanism of AZ91 Mg alloys in a dynamic marine atmosphere, which is of great significance for promoting the application of Mg alloys in marine engineering.

## 2. Materials and Methods

### 2.1. Material Preparation

The chemical composition of as-extruded AZ91 Mg alloy is listed in Table 1. The size of the experimental samples was 100 mm × 50 mm × 3 mm. All specimens were ground with successive grades of SiC grit paper from #400 to #1000. The specimens were cleaned using distilled water and degreased with acetone, dried with cool air, and stored in a desiccator. Five parallel samples were exposed for each period. Three samples were used to determine the weight loss of specimens and the remaining were used for corrosion performance evaluation.

### 2.2. Atmospheric Exposure Experiment

**Figure 1:**
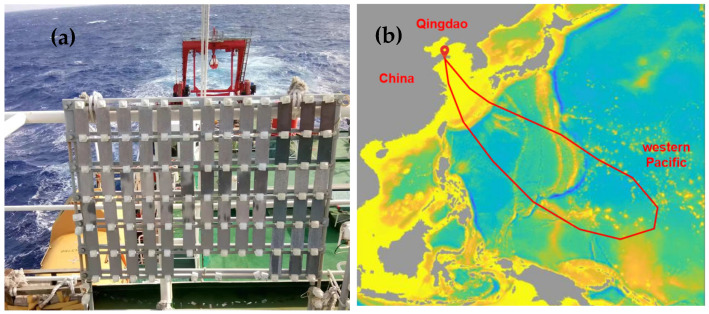
Atmospheric environmental corrosion test on Research Vessel KEXUE (**a**) exposure rack, (**b**) navigation route.

The dynamic atmospheric exposure experiment was carried out on the deck of the Research Vessel KEXUE (Science) of the Institute of Oceanology, Chinese Academy of Sciences (Qingdao, China). AZ91 magnesium alloy was placed on the exposure rackwith an angle of 45° horizontal to the deck for one year (Figure 1a). The sailing route was from Yellow Sea (Qingdao, China) to Western Pacific Ocean (Figure 1b), which spanned from a northern temperate climate to a tropical climate. The exposure period was from September 2020 to September 2021 (5 ocean voyages were conducted), with four intervals at 1, 3, 6, and 12 months.

### 2.3. Environmental Factors Measurements

The meteorological factors (such as temperature and relative humidity (RH)) were measured by the automatic weather observation system of Chinese Research Vessel KEXUE (Science).

The deposition rate of chloride was measured based on GJB 8894.1-2017 [21]. Three parallel specimens of double-layer gauze with size of 100 cm^2^ were exposed under the rain shelter on the deck for 7 days. The collected gauze specimens were cleaned, and the chloride ion concentration was measured using an Ion-Chromatography instrument (ICS-5000, ThermoFisher, Waltham, MA, USA).

**Figure 2:**
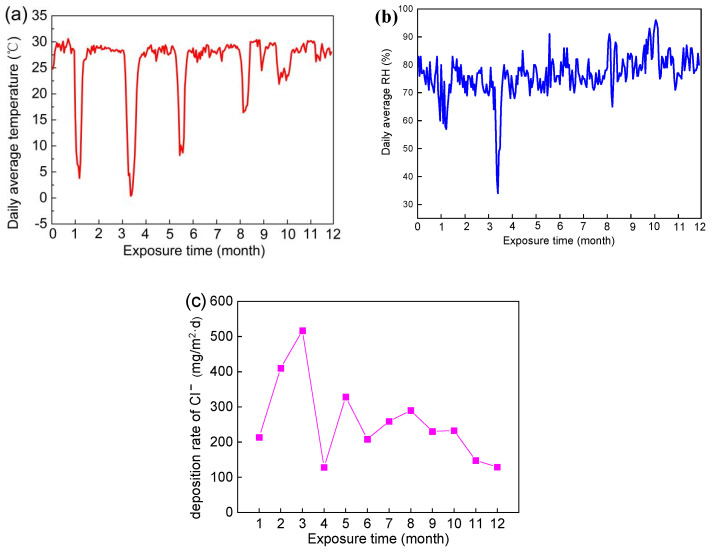
Environment factor of exposure site during the exposure time: (**a**) temperature, (**b**) relative humidity, and (**c**) chloride ion deposition rate [17].

The data for temperature, relative humidity, and chloride ion deposition rate during a one-year sea voyage are shown in Figure 2.

### 2.4. Characterization and Analysis of the Exposed Samples

The surface and cross-section morphology of the corroded samples was observed by scanning electron microscope (SEM, Regulus 8100, HITACHI, Tokyo, Japan), energy dispersive spectrometer (EDS), metallographic microscope (Axio Vert.A1, Hanover, Germany), and laser confocal scanning microscopy (LCSM, OLS5000, Olympus, Tokyo, Japan). Phase composition was analyzed by X-ray diffraction (XRD, Ultime IV, Rigaku, Tokyo, Japan) with a Cu target and a monochromator, at 40 kV and 150 mA with a scanning rate of 10°/min and a step size of 0.02°. The element types and valence states of the corrosion products were analyzed by X-ray photoelectron spectroscopy (XPS, ESCALAB 250Xi, Thermo, Waltham, MA, USA).

The corrosion products were removed using 200 g/L CrO_3_ + 10 g/L AgNO_3_ immersed for 5–10 min at 25 °C, and then the samples were rinsed with distilled water and alcohol, dried for 24 h, and weighted. The samples before and after exposure were weighed using an analytical balance with an accuracy of 0.1 mg.

The corrosion rate of AZ91 magnesium after exposure for different duration was calculated using the equation as follows:

*v* = (*w*_0_ − *w*_1_)/(*S*·*T*·*ρ*) (1)

In the above formula, *v* is the corrosion rate of AZ91 Mg alloys. *w*_0_ and *w*_1_ are the initial and final mass (after removing the corrosion products), respectively. *S* represents the surface area. *T* is the exposure time, and *ρ* is the density of AZ91 Mg alloy.

## 3. Results and Discussion

### 3.1. Initial Microstructure of AZ91 Mg Alloy

**Figure 3:**
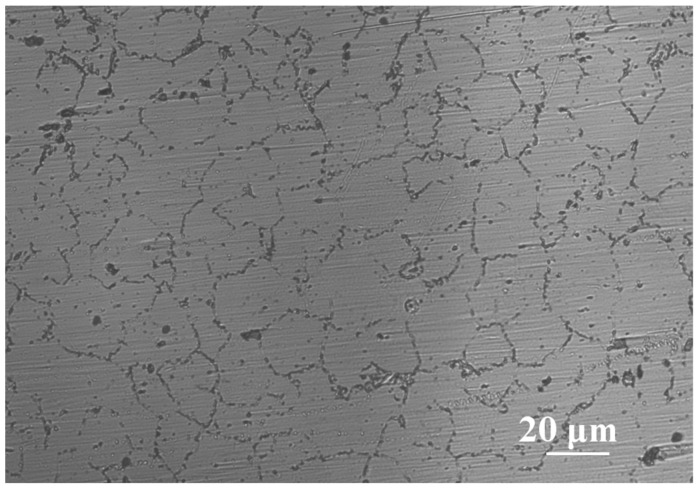
Optical micrograph of AZ91 Mg alloy.

Figure 3 shows the optical micrograph of AZ91 Mg alloy. The image clearly exhibits the α-Mg matrix and β-phase interdendritic network. As is well known, the Al content of AZ91 Mg alloy exceeds its solubility limit in magnesium, and β-phase Mg_17_Al_12_ intermetallic compounds are formed near grain boundaries during solidification [22,23].

### 3.2. Corrosion Morphologies of AZ91 Mg Alloys Exposed to Dynamic Marine Atmosphere

**Figure 4:**
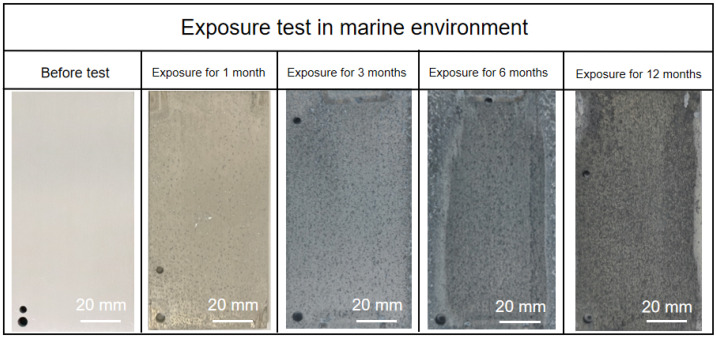
Macroscopic images of AZ91 magnesium alloy specimens exposed for different periods during an ocean-going voyage.

The macroscopic images of AZ91 magnesium alloy specimens with corrosion products exposed for different periods during an ocean-going voyage are shown in Figure 4. It can be clearly seen that the color of the surface gradually becomes darker with the increase in exposure time. Due to the increasing number of corrosion pits and formation of corrosion products, the surface of magnesium alloy gradually became rough and lost its metallic luster after exposure for 3 months. There are obvious traces of rain erosion on the surface of the samples after exposure for 6 months and 12 months, which may lead to some loose corrosion products and soluble compounds being washed away.

**Figure 5:**
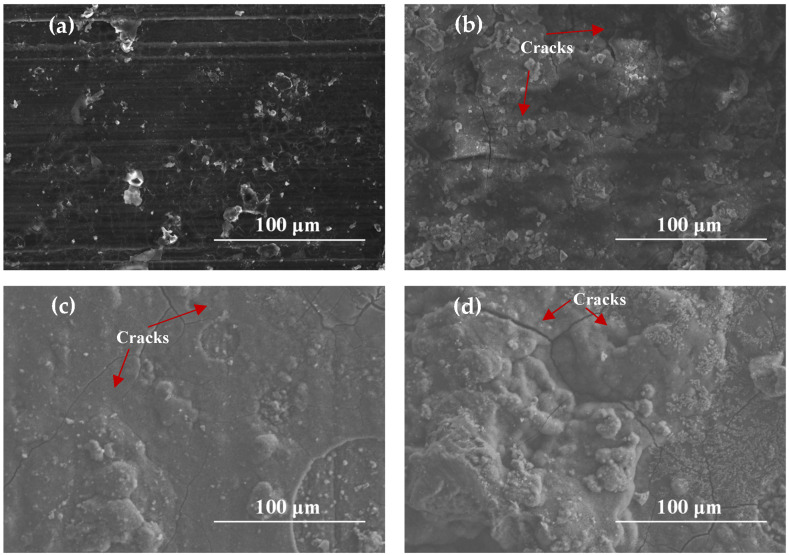
SEM images of AZ91 Mg alloy specimens exposed for different periods: (**a**) 1 month, (**b**) 3 months, (**c**) 6 months, (**d**) 12 months.

Figure 5 exhibits SEM images of AZ91 magnesium alloy specimens exposed for different periods during an ocean-going voyage. As shown in Figure 5a, some randomly distributed corrosion products formed on the surface of the sample after one month of exposure. The corrosion film was not compact, and many cracks and scratches on the surface of the substrate can be clearly seen. When the Mg alloy samples had been exposed for 3 to 12 months, it can be seen that the morphologies of the corrosion products were similar. The number of corrosion products gradually increased, and there were many cracks that may cause the infiltration of corrosive media such as chloride ions. In some areas, the corrosion products appeared as flower clusters, which were loose and easily washed away by rainwater or strong winds.

**Figure 6:**
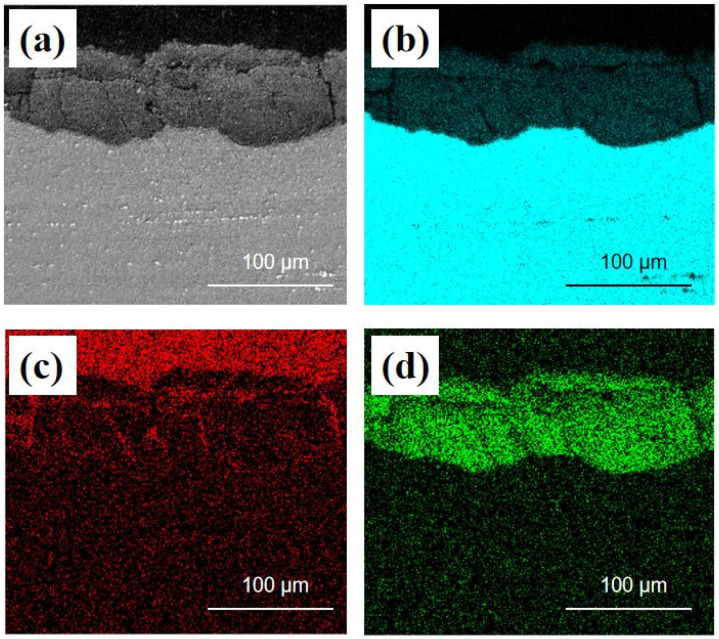
EDS analysis of the cross-section of AZ91 magnesium alloy exposed for 1 year on the deck of Research Vessel KEXUE: (**a**) electron image, (**b**) Mg map, (**c**) C map, (**d**) O map.

Figure 6 displays the EDS analysis of the cross-section of AZ91 Mg alloy exposed for 1 year on the deck of the Research Vessel KEXUE. After exposure for 12 months, the corrosion product layer with a thickness of more than 90 µm formed on the surface of the samples. The O element was mainly concentrated on the corrosion product layer, which indicated that the corrosion product layer formed on AZ91 magnesium alloy was composed of oxide. It can also be seen that there were some small cracks in the corrosion product layer, and the C element was concentrated in these cracks. This is mainly due to the thin electrolyte layer formed on the surface of specimens. CO_2_ in the air and dissolved in the thin electrolyte layer could permeate into these cracks and generate carbonate-containing compounds [24,25]. In high relative humidity environments, the electrolyte solution formed on the surface could penetrate into the substrate surface through cracks, promoting further corrosion.

**Figure 7:**
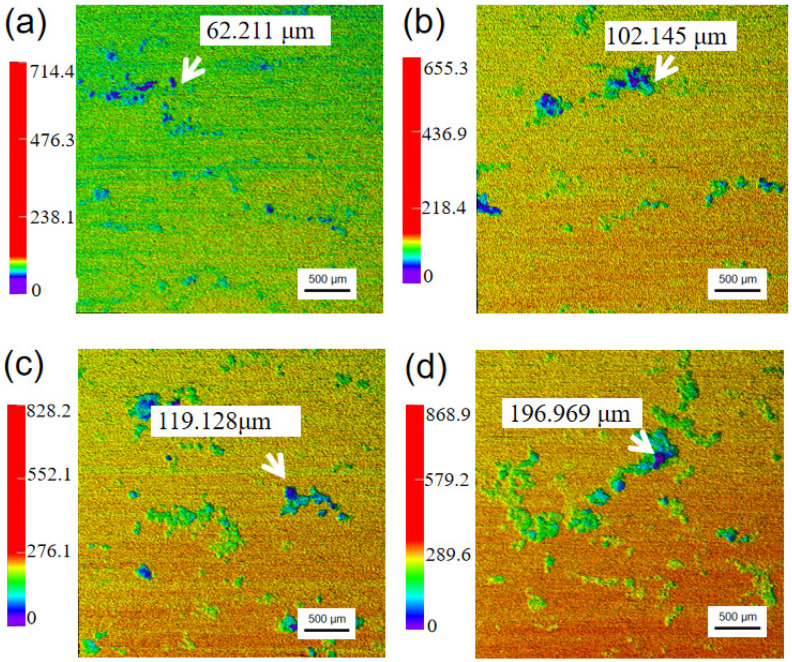
Laser confocal scanning microscopy (LCSM) of AZ91 magnesium alloy specimens without corrosion products exposed for different periods: (**a**) 1 month, (**b**) 3 months, (**c**) 6 months, (**d**) 12 months.

Figure 7 displays laser confocal scanning microscopy (LCSM) analysis of AZ91 magnesium alloy specimens exposed for different periods in a marine environment. It can be obviously seen that the number of pitting corrosions and the maximum pitting depth gradually increase with the increased exposure time. After 12 months of exposure, the maximum pitting depth was approximately 197 µm, which is about 6 times that of the average corrosion depth. Combined with the analysis of morphology of the corrosion products, the corrosion pits formed on the specimens may be related to the thin electrolyte layer, which contained a high concentration of chloride ions. The thin electrolyte layer having a high concentration of chloride ions might permeate into the matrix through cracks and react with the matrix, causing serious localized corrosion in these areas.

### 3.3. Corrosion Products Analysis

**Figure 8:**
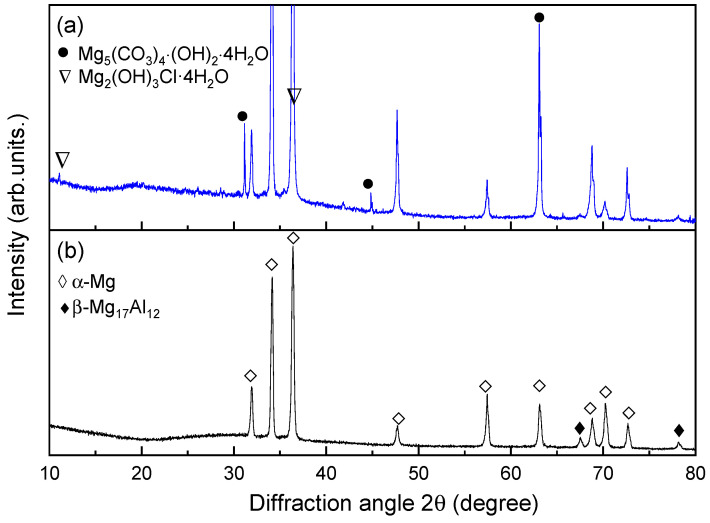
XRD patterns of AZ91 magnesium alloy with: (**a**) corrosion products, (**b**) matrix.

Figure 8 exhibits the XRD patterns of AZ91 magnesium alloy with corrosion products and matrix. The results show that the corrosion products on the surface of AZ91 magnesium alloys exposed in marine environments for 12 months were mainly composed of hydromagnesite (Mg_5_(CO_3_)_4_(OH)_2_·4H_2_O [26] and the chloride-containing compound Mg_2_(OH)_3_Cl·4H_2_O [27]. In previous reports on the corrosion products of specimens exposed to different atmospheric environments, Mg_5(_CO_3_)_4_(OH)_2_·4H_2_O was found together with MgCO_3_ and/or MgCO_3_·xH_2_O (x = 3, 5) [12,14]. However, there are no obvious peaks of MgCO_3_ detected in the XRD analysis of this work, indicating that it may be low content or amorphous structures.

**Figure 9:**
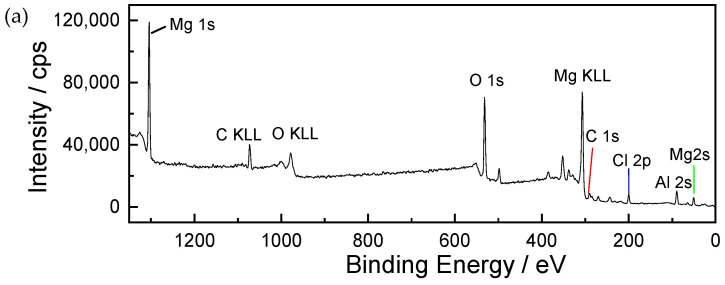

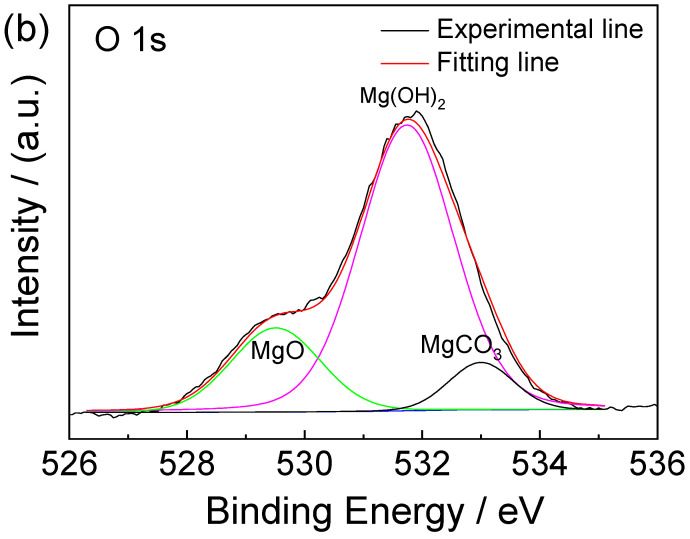
The XPS binding energy spectrum of corrosion products formed on AZ91 magnesium alloy exposed for 12 months in the marine environment of an ocean voyage: (**a**) whole spectrum, (**b**) narrow scan spectrum of O 1s. Different colors were used to distinguish different binding states of oxygen element.

Figure 9 shows the XPS spectrum of corrosion products formed on AZ91 magnesium alloy exposed for 12 months in the dynamic marine atmospheric environment ((a) the whole spectrum, (b) narrow scan spectrum of O 1s). The results show that the main elements of the surface contained a large amount of Mg, O, and C, and a small amount of Al and Cl, as shown in Figure 9a. In Figure 9b, the O 1s spectrum consists of three main peaks. The peaks at 529.5 eV and 531.8 eV are respectively associated with MgO and Mg(OH)_2_ [28,29]. Furthermore, the peak present on the O 1s high-resolution spectrum at 533 eV is ascribed to MgCO_3_, a compound that is also commonly found in the Mg corrosion layer [30].

### 3.4. Corrosion Rate of AZ91 Mg Alloy Exposed to Dynamic Marine Atmosphere for Different Durations

**Figure 10:**
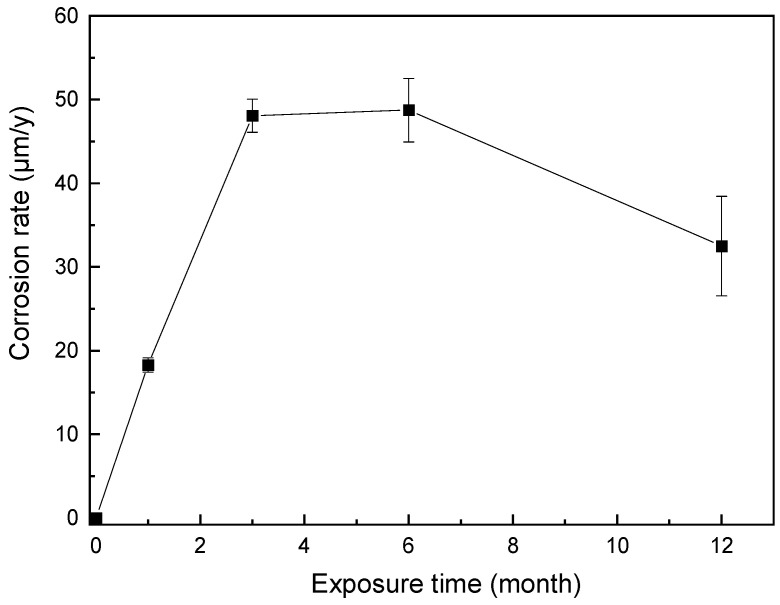
Variation in corrosion rate of AZ91 Mg alloy exposed to a dynamic marine atmosphere for different durations.

The variation curve of the corrosion rate of AZ91 Mg alloy on the deck of Research Vessel KEXUE after 1 year of exposure is shown in Figure 10. In the first three months of exposure, the corrosion rate rapidly increased with an approximately consistent slope. The corrosion rate in three months reached as high as 48.08 μm/y. The corrosion rate remained stable for the next three months, and after six months of exposure, the corrosion rate of the sample gradually decreased with the passage of exposure time. After 12 months of exposure, the corrosion rate of the sample was 32.50 μm/y. The reason for this is speculated to be the more positive potential of the second phase (β-Mg_17_Al_12_) compared to the matrix phase [31], resulting in micro-galvanic corrosion. After 3 months, the micro-galvanic corrosion and the hindrance of the second phase reached equilibrium, and the corrosion rate remained unchanged. After 6 months, with the dissolution of the matrix phase, the relative proportion of the second phase increased, and its hindrance became more obvious. The thick corrosion product film also hindered the infiltration of chloride ions, resulting in decreasing the corrosion rate of AZ91 Mg alloy.

The corrosion rate of AZ91 magnesium alloy exposed on the deck of Research Vessel KEXUE was five to eight times that of the investigations conducted in static marine environments, such as the study conducted in a marine environment located 3–5 m away from the Atlantic shore, Brest, France [14], and the research conducted in a marine environment 600 m from the coast in Xiamen, China [15]. The more severe marine atmospheric corrosion behavior of AZ91 magnesium alloy may be due to the special exposure environment from the Yellow Sea to the Western Pacific Ocean, including rapid temperature changes, high humidity, and chloride ion deposition rate.

Compared with exposure tests conducted in static marine atmospheric environments, the temperature shows seasonal changes with changes in longitude and latitude in a real dynamic marine atmospheric environment. Sudden alternating changes in temperature may increase the cracks in corrosion products, leading to the infiltration of corrosive media such as chloride ions and accelerating the occurrence of corrosion [13]. During the exposure period, the marine scientific research vessel carried out five voyages from the Yellow Sea to the Western Pacific Ocean. After exposure for 3 months, the Research Vessel KEXUE returned to Qingdao from the Western Pacific Ocean. The average temperature in the Western Pacific Ocean was maintained at about 29 °C all year round. There were five sudden drops in temperature during the exposure period (Figure 2a), which were closely related to the voyages returning to the Yellow Sea.

The deposition rate of chloride is extremely high in a real dynamic marine atmospheric environment. During the ocean voyage, the monthly deposition rate of chloride was above 100 mg/m^2^·d, and specifically above 1100 mg/m^2^·d in the third month (Figure 2c). As is well known, NaCl has a strong corrosive effect on magnesium alloys, which is closely related to the high corrosion rate of magnesium alloys in dynamic marine atmospheric environments [17].

It was worth noting that relative humidity (RH) was high and, during the exposure period, the proportion of time when RH > 75% was 56% during the ocean voyage. Previous reports have revealed that NaCl can form a salt solution by absorbing water at relative humidity > 75% [32], which indicates a thin electrolyte layer having a high concentration of chloride could form on the surface of specimens for more than half of the sailing time. A thin electrolyte layer provides a near-solution environment for electrochemical corrosion, leading to large-scale electrochemical connections on the surface and increasing the corrosion process of Mg alloy. Therefore, AZ91 magnesium alloy suffers severe corrosion in real dynamic marine environments due to the special environmental conditions noted above.

### 3.5. Corrosion Mechanism of AZ91 Mg Alloy in Dynamic Marine Atmospheric

**Figure 11:**
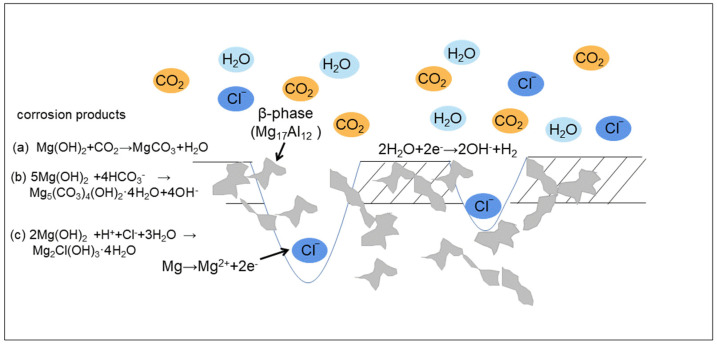
The corrosion process schematic of AZ91 magnesium alloy.

Figure 11 shows the corrosion process schematic of AZ91 magnesium alloy during exposure in the dynamic marine atmosphere.

Due to the high deposition rate of chloride and high RH, AZ91 magnesium alloy was covered by a thin electrolyte layer having a high concentration of Cl^−^ when exposed in a real dynamic marine atmosphere. The corrosion of AZ91 magnesium alloy was dominated by the chemical reaction process including oxidation and hydration reactions at the initial stage.

Anodic reaction:

Mg → Mg^2+^ + 2e^−^ (2)

Cathodic reaction:

2H_2_O + 2e^−^ → 2OH^−^ + H_2_ (3)

Because of the existence of the thin electrolyte layer, Mg(OH)_2_ easily formed on the surface of the specimens [33].

Brucite reacted with CO_2_ (which came from the marine atmosphere and gas generated by the combustion of fuel) to form MgCO_3_ as follows [24]:

Mg(OH)_2_ + CO_2_ → MgCO_3_ + H_2_O (4)

CO_2_ reacted with H_2_O to form HCO_3_^−^, and then reacted with brucite [19]:

5Mg(OH)_2_ + 4HCO_3_^−^ → Mg_5_(CO_3_)_4_(OH)_2_·4H_2_O + 4OH^−^ (5)

Brucite reacted with H^+^ (derived from the dissolution of acidic gases such as SO_2_ from fuel combustion in thin liquid films), Cl^−^, and H_2_O to form Mg_2_Cl(OH)_3_ as follows [25]:

2Mg(OH)_2_ + H^+^ + Cl^−^ + 3H_2_O → Mg_2_Cl(OH)_3_·4H_2_O (6)

A corrosion product layer formed on the surface of specimens experiencing a rapid change in temperature during the ocean-going voyage. The volume changes in the matrix and the corrosion product layer were different when temperature changed rapidly. Therefore, there was obvious stress at the interface between the matrix and the corrosion product layer, which accelerated the detachment of corrosion products and generation of cracks. The thin electrolyte layer having a high concentration of chloride ions could permeate into the matrix through these cracks and react with matrix, causing severe localized corrosion. The corrosion behavior of AZ91 Mg alloy was still dominated by localized corrosion after exposure for 12 months, which was mainly due to micro-galvanic corrosion between the α-matrix and β-phase.

Earlier research reported that the β-phase has more positive potential than the α-Mg matrix. When α-Mg is dissolved, the β-phase still remains on the surface. Mg_17_Al_12_ precipitates with high connectivity along the grain boundary and inside the alloy grains, functioning as a barrier to inhibit the corrosion.

## 4. Conclusions

The corrosion rate of AZ91 Mg alloy during navigation from the Yellow Sea to the Western Pacific Ocean can be divided into three stages: rapidly increasing in the first three months, then remaining almost the same for the next three months, and ultimately significantly decreasing after 6 months of exposure. The corrosion rate was 32.50 μm/y after 12 months of exposure in a dynamic marine atmospheric environment during an ocean voyage, which is 5–8 times higher than that of static coastal field exposure.

In the studied dynamic marine atmospheric environment, AZ91 magnesium alloy was mainly subjected to localized corrosion. After 12 months of exposure, the maximum depth of the corrosion pit was 197 μm, which was almost 6 times that of the average corrosion depth. Localized corrosion poses greater risks to the service life of magnesium alloys. The pollutants of NaCl particles accelerate the localized corrosion with the coupling effect of meteorological factors and the microstructure of the AZ91 Mg substrate.

This study provides scientific data for the application of magnesium alloy in shipboard aircraft and other equipment. It is expected that this research will be of benefit to engineers and researchers developing new corrosion-resistant technology for marine applications.

## Figures and Tables

**Table 1:** Chemical composition of AZ91 magnesium alloy (wt.%).

Material	Al	Zn	Mn	Si	Fe	Cu	Ni	Mg
AZ91	8.93	0.68	0.25	0.02	0.003	0.003	0.0006	Bal.

## Data Availability

Data are contained within the article.
